# Targeted next-generation sequencing-based sequencing of cell-free DNA in cerebrospinal fluid uncovers cancer-specific mutations in patients with brain cancer using a widely available panel

**DOI:** 10.1093/noajnl/vdaf270

**Published:** 2026-01-07

**Authors:** Alexander Köpp, Peter Westarp, Marta Nowak, Nils Briel, Maximilian Mastall, Adela Brzobohata, Roger Schenk, Michael Schmid, Emilie Le Rhun, Holger Moch, Michael Weller, Martin Zoche, Tobias Weiss

**Affiliations:** Department of Neurology, Clinical Neuroscience Center, University Hospital Zurich and University of Zurich, Zurich, Switzerland; Department of Pathology and Molecular Pathology, University Hospital Zurich and University of Zurich, Zurich, Switzerland; Department of Medical Oncology and Hematology, University Hospital Zurich, Zurich, Switzerland; Department of Neurology, Clinical Neuroscience Center, University Hospital Zurich and University of Zurich, Zurich, Switzerland; Department of Neurology, Clinical Neuroscience Center, University Hospital Zurich and University of Zurich, Zurich, Switzerland; Department of Neurology, Clinical Neuroscience Center, University Hospital Zurich and University of Zurich, Zurich, Switzerland; Department of Pathology and Molecular Pathology, University Hospital Zurich and University of Zurich, Zurich, Switzerland; Department of Pathology and Molecular Pathology, University Hospital Zurich and University of Zurich, Zurich, Switzerland; Department of Medical Oncology and Hematology, University Hospital Zurich, Zurich, Switzerland; Department of Pathology and Molecular Pathology, University Hospital Zurich and University of Zurich, Zurich, Switzerland; Department of Neurology, Clinical Neuroscience Center, University Hospital Zurich and University of Zurich, Zurich, Switzerland; Department of Pathology and Molecular Pathology, University Hospital Zurich and University of Zurich, Zurich, Switzerland; Department of Neurology, Clinical Neuroscience Center, University Hospital Zurich and University of Zurich, Zurich, Switzerland

**Keywords:** cell-free DNA, cerebrospinal fluid, liquid biopsy, targeted sequencing

## Abstract

**Background:**

The gold standard for the diagnosis of brain tumors comprises neurosurgical biopsies or resections for tissue acquisition. Depending on the anatomical location of the tumor, this might not always be feasible. The aim of this study was to assess whether targeted sequencing of cell-free DNA (cfDNA) could be applied in a clinical setting for the detection of cancer-specific mutations in cerebrospinal fluid (CSF) and blood using a widely available panel, investigating if this approach would facilitate brain tumor diagnosis.

**Methods:**

Patients with newly suspected or confirmed CNS tumors or suspected leptomeningeal metastasis were included. CSF and blood were sampled for each patient. Using next-generation sequencing (NGS), targeted sequencing was performed on cfDNA. If available, matched tissue samples were also sequenced using the same gene panel.

**Results:**

We investigated 58 samples from 29 patients. Sequencing was successful in 16 CSF samples (55%) and 29 blood samples (100%). On average, more tumor-specific mutations were found in CSF compared to blood. Patients in whom cfDNA sequencing in CSF was successful had tumors with a significantly shorter distance to the CSF space and were larger in size. In 2 cases, where surgeries were not considered because of high surgical risk due to the anatomical locations of the lesions, the diagnosis was aided by CSF sequencing results. One primary CNS lymphoma and 1 H3 K27M diffuse midline glioma were identified, respectively.

**Conclusions:**

NGS-based targeted sequencing in CSF has the potential to facilitate brain tumor diagnosis in patients presenting with new cerebral lesions.

Key points

- Next-generation sequencing-based targeted sequencing of cell-free DNA (cfDNA) is feasible in cerebrospinal fluid (CSF) with a success rate of 55%.
- Patients in whom cfDNA sequencing in CSF was successful had tumors with a significantly shorter distance to the CSF space and that were larger in size.
- Results from cfDNA sequencing in CSF in patients with brain cancer are more informative than from blood. In 2 cases, the diagnosis could be aided by detecting cancer-specific mutations in CSF.

Importance of the Study

Malignant brain tumors are among the most challenging malignancies. A fast and accurate diagnosis is necessary to offer the most appropriate treatment. Depending on the location, tissue sampling might not always be possible or is associated with high risks for neurological deterioration. We performed next-generation sequencing-based targeted sequencing of cell-free DNA in cerebrospinal fluid (CSF) and blood using a research-use-only assay based on the FoundationOne Liquid CDx test. We included patients with new cerebral lesions as well as patients undergoing lumbar puncture for suspected leptomeningeal metastasis or known cerebral tumors. Overall, more cancer-specific mutations were detected in CSF than in blood. In 2 cases, the diagnosis was aided by the sequencing results. This underscores its potential added value when integrated into the clinical workflow.

Brain cancer is still one of the most challenging malignancies. An accurate diagnosis is important to select the most appropriate treatment for each patient and is based on histopathological and molecular analysis of tumor material according to the 2021 WHO classification of CNS tumors.^1^ Currently, this requires tissue sampling of the tumor. However, surgical procedures like biopsies or resections have a perioperative risk and specific limitations in the CNS. Stereotactic biopsies are associated with an inpatient mortality of 2.8%.^2^ Especially in cases where the tumor is located in eloquent regions like the brain stem, a biopsy might not be considered feasible.

Recent technological advances have demonstrated the ability to detect cancer-specific mutations in cell-free DNA (cfDNA). Cell-free DNA is shed into the blood or cerebrospinal fluid (CSF) when cells die.^3^ Previous studies used digital droplet PCR to detect hot-spot mutations like the isocitrate dehydrogenase 1 mutation in cfDNA in blood or other tumor-defining mutations like the *H3* K27M mutation for diffuse midline glioma in serial CSF samples.^4^^,^^5^ In a multiplexed approach, selected genes, including *MYD88*, were analyzed for the detection of CNS lymphoma in CSF.^6^ Other groups have created in-house next-generation sequencing (NGS) panels for the molecular-guided classification of CNS tumors based on single nucleotide variants, copy number variations, or detection of insertions and deletions in the CSF.^7-10^ Compared with peripheral blood, CSF is the preferred analyte due to its proximity to the tumor in patients with brain cancers.^11^

The FoundationOne Liquid CDx (F1LCDx) is an NGS-based gene panel for genomic profiling of tumor-derived cfDNA in blood.^12^^,^^13^ However, the applicability to brain tumors and CSF is unknown. In the present study, we investigated the performance of this NGS-based gene panel for the genomic profiling of cfDNA extracted from CSF and blood in a clinical setting in patients with cerebral lesions.

## Methods

### Patient Cohort

Patients with a new cerebral lesion as examined by MRI or suspicion of leptomeningeal metastasis or known cerebral tumors with a clinically indicated CSF analysis were approached for informed consent from 2023 to 2025. Written consent was obtained before the sampling of blood or CSF. The study was conducted in accordance with the World Medical Association Declaration of Helsinki, and approval by the Institutional Review Board was obtained (BASEC-Number: 2021-00652). Exclusion criteria were age under 18 years or contraindications to lumbar puncture (eg anticoagulation, platelet count <50 G/l, risk of herniation).

### CSF and Blood Collection

Additional CSF was collected at the occasion of a clinically indicated lumbar puncture according to the standard operating procedure of the Department of Neurology at the University Hospital Zurich. For both blood and CSF, the same cfDNA collection tubes were used (CE-IVD Cell-Free DNA Collection Tube, Roche, Basel, Switzerland). For each patient, a minimum of 2.5 mL and a maximum of 10 mL CSF was collected. Peripheral blood was obtained via venipuncture collecting 16 mL. As per clinical routine, CSF chemistry (glucose, lactate) and CSF/blood albumin ratios were measured. Blood was centrifuged the same day of venipuncture at 2000*g* for 15 min to separate plasma and was stored at 4 °C.

### DNA Extraction

For isolation of cfDNA from blood and CSF, the MagMAX Cell-Free DNA Isolation Kit (ThermoFisher, Waltham, MA) on a KingFisher system (ThermoFisher, Waltham, MA) and a single extraction method were used according to the manufacturer’s protocol. For genomic DNA extraction from formalin-fixed paraffin-embedded tissue, the Maxwell RSC DNA FFPE Kit (Promega) on a Maxwell RSC instrument (Promega) was used following the manufacturer’s protocol.

### Molecular Analysis

Liquid biopsy specimens from plasma and CSF were analyzed using a research-use-only assay based on F1LCDx (Foundation Medicine, Inc., Cambridge, MA), an NGS-based in vitro diagnostic assay. This assay employs the whole-genome shotgun method for library construction and targeted high-throughput hybridization-based capture technology to detect and report alterations in 324 genes, as well as ctDNA tumor fraction and genomic signatures, including microsatellite instability and blood tumor mutational burden. Following hybrid capture, the libraries were sequenced with deep coverage using the Illumina NovaSeq 6000 platform. It is important to note that this assay has not been designed for CSF analysis, and the application to CSF samples in the present study does not represent the intended use of the F1LCDx test. Bioinformatic analysis, including detection of genomic alterations, that is, base substitutions, indels, selected copy number variants (gene amplifications and homozygous deletions), and selected genomic rearrangements as well as biomarkers, was performed using a proprietary customized analysis pipeline developed by Foundation Medicine, Inc. Successful sequencing was defined, among other criteria, as achieving a predefined minimum coverage threshold across the panel, ensuring sufficient depth for reliable variant calling. Fragment length of cfDNA was calculated from mapped sequencing data as the insert size, that is, the length of the DNA fragment between adapters. The details on the sequencing and analytical validation of the assay have been published previously.^12^ Clinical interpretation of sequencing results was restricted to established brain tumor mutations, excluding isolated variants in genes frequently associated with clonal hematopoiesis.

### Tumor Radiological Characteristics

To approximate the tumor size, the product of diameter (POD, largest perpendicular diameter) was calculated using contrast-enhanced, T1-weighted MRI. The MRI scan closest in time to the lumbar puncture was selected for analysis. If the cerebral lesion did not show any contrast enhancement, the patient was not further analyzed regarding tumor size.

The distance from the tumor to the CSF space was defined as the shortest distance of the contrast-enhancing tumor to the CSF on contrast-enhanced, T1-weighted MRI. Patients with newly diagnosed brain lesions, which were diagnosed later as non-neoplastic, were excluded for this analysis.

### Statistical Methods

All statistical analysis were performed in GraphPad Prism (Version 10.3.1). Normal distribution was checked using Shapiro–Wilk test for group-wise comparisons of continuous variables. For normally distributed variables, we used a 2-sided *t*-test. For non-normally distributed variables, we applied the non-parametric Mann–Whitney *U*-test, unless otherwise stated. Statistical significance was set to *P* < .05.

## Results

### NGS-Based cfDNA Sequencing in CSF is Feasible

Twenty-nine patients with new cerebral lesions, a suspicion of leptomeningeal metastasis, or known cerebral tumors were included (Figure 1A; Table 1; Table S1). Sequencing of cfDNA in CSF was successful in 16 patients (55%) (Figure 1B). In contrast, in blood, it was successful in all samples (*N* = 29, 100%). To investigate confounding variables that could explain the success of cfDNA sequencing in CSF, we considered several factors, such as total CSF volume retrieved, DNA amount, or tumor size. Regarding collected CSF volumes, there were no significant differences between the groups with successful sequencing versus failed sequencing (*P* = .74). The mean volume was 7.03 ± 2.11 mL in the successful sequencing group and 6.79 ± 1.4 mL in the failed sequencing group (Figure 1C). While the extracted DNA amount was not significantly different (9.01 ± 8.14 ng vs. 6.47 ± 3.71 ng, *P* = .56), it should be noted that the accepted cfDNA input for the assay is specified as 20 ng. Only after hybrid capture, the DNA yield in the successful sequencing group was higher than that in the failed sequencing group (*P* = .003, Figure S1A). Notably, patients for whom the sequencing of cfDNA from the CSF was successful had a contrast-enhancing tumor closer to the CSF than those for whom sequencing was not successful (0.39 ± 1.07 mm vs. 3.13 ± 2.64 mm, *P* = .0008) (Figure 1D). In addition, the tumor size approximated by the POD was larger in the successful sequencing group (899.1 ± 915.6 mm^2^ vs. 148.3 ± 99.48 mm^2^, *P* = .0027, Figure 1D). Furthermore, glucose levels, lactate levels, and the albumin ratio were not significantly altered between these 2 groups (Figure S1C).

**Figure 1.** Targeted NGS of cfDNA in CSF versus peripheral blood. (A) Workflow of the presented study. (B) Ratio of successful cfDNA sequencing from CSF. (C) Left: CSF volume in mL after lumbar puncture for the NGS successful versus NGS failed groups (*P* = .74). Right: DNA yield in ng for these groups (*P* = .56). (D) Left: Distance to CSF in mm measured by contrast-enhancing tumor using contrast-enhanced, T1-weighted MRI (*P* = .0008). Right: Tumor size measured by product of diameter (POD) in mm^2^ of contrast-enhancing lesion on T1-weighted MRI (*P* = .0027). NGS, next-generation sequencing; cfDNA, cell-free DNA; CSF, cerebrospinal fluid **<0.01 ***<0.001.

**Table 1.** Clinical characteristics of included patients

	Overall (*N* = 29)
**Age (years)**	Mean: 55.4
**Sex**	
Male	15 (51.7%)
Female	14 (48.3%)
**Disease entity**	
AEG	1 (3.4%)
Astrocytoma, high grade	1 (3.4%)
Breast cancer	5 (17.2%)
Germinoma	2 (6.9%)
Glioblastoma	5 (17.2%)
LPD	1 (3.4%)
Lung cancer	3 (10.3%)
Lymphoma	1 (3.4%)
Melanoma	4 (13.8%)
non-neoplastic	3 (10.3%)
Unknown	3 (10.3%)
**Blood analysis**	
Failed	0 (0%)
Successful	29 (100%)
**CSF analysis**	
Failed	13 (44.8%)
Successful	16 (55.2%)

Abbreviations: AEG, esophagogastric junctional adenocarcinoma; CSF, cerebrospinal fluid; LPD, lymphoproliferative disorders; unknown, no final diagnosis available.

### cfDNA Sequencing Was More Informative in CSF than in Blood in This Cohort

We sequenced CSF and blood for each patient. The average length of cfDNA fragments was 167 bp in blood, whereas the signal from cfDNA from CSF showed several peaks, with the most frequent at 167 and 143 bp (Figure S2). On average, we found 17 mutations, including known pathological mutations and variants of uncertain significance, in the CSF compared to 8 in the blood (Figure 2A). In the CSF, 3 known pathological mutations were found on average compared to 1 in the blood. Next, we determined which genetic profiles were revealed by NGS-based cfDNA sequencing in patients with glioblastoma and compared the liquid-based sequencing to tissue-based sequencing results. For 3 patients, we had matched tumor tissue available (Figure 2B). Most mutations found within the tissue were also found in cfDNA in the CSF, but not in the blood. Importantly, a tissue-confirmed *BRAF* V600E mutation was only detected in the CSF, but not in the blood. Other tissue alterations like *CDKN2A* loss, *CDKN2B* loss, or *MTAP* loss were neither detected on cfDNA in CSF nor in the blood. Twenty-two known pathogenic mutations were found in tissue and CSF combined, of which 12 (55%) were shared between tissue and CSF (Figure 2C). In contrast, only 4 of 20 (18%) known pathogenic mutations were shared between tissue and blood in these 3 glioblastoma patients.

**Figure 2.** Sequencing results of cfDNA in CSF and blood and comparison to tissue. (A) Left: Mutations found in blood and CSF (known pathological mutations and variants of uncertain significances [VUS]). Each point represents 1 patient. Right: Reduced to known pathological mutations detected in serum and CSF. (B) For *N* = 3 patients with known glioblastoma-matched tissue was available. The plot shows which mutations were shared or private between tissue, CSF, and serum. White squares indicate that the alteration was not present in this patient. (C) Pie charts showing the distribution of shared or private mutations among all identified variants between each 2 of CSF, serum, and tissue of the same 3 patients with glioblastoma. In the lower pie chart of CSF and serum, the results of *N* = 5 patients with known glioblastoma are shown. cfDNA, cell-free DNA; CSF, cerebrospinal fluid.

### cfDNA Sequencing in CSF Enables the Diagnosis of New Cerebral Lesions

Within this study, we also included patients with a suspicion of brain tumor. Patient 19 was a 74-year-old male patient presenting with progressive reduced alertness and confusion. The brain MRI revealed a diffuse contrast-enhancing parietal mass close to the ventricle (Figure 3A). Sequencing of cfDNA from CSF showed a *MYD88* mutation with a variant allele frequency (VAF) of 18.37% (Figure 3B;  Figure S3). The mutation was not detected in cfDNA from blood. Due to the rapid decline of the patient, a biopsy was performed, which later confirmed the *MYD88* mutation leading to the diagnosis of a primary CNS lymphoma (Figure 3C).

**Figure 3.** Sequencing of cfDNA in CSF enables diagnosis of brain cancer. (A) Contrast-enhanced, T1-weighted MRI of patient 19 and patient 36 with new cerebral lesions. (B) Results of cfDNA sequencing of patients 19 and 36 in CSF and blood. The known pathological mutations are marked in yellow, in white VUS. (C) Showing the results of tissue sequencing from a biopsy of patient 19. cfDNA, cell-free DNA; CSF, cerebrospinal fluid; VUS, variants of uncertain significance.

Patient 36 was an 88-year-old male patient. The MRI showed several contrast-enhancing lesions in the brain stem close to the fourth ventricle and the aqueduct (Figure 3A). A biopsy was not recommended by the interdisciplinary tumor board due to the associated risks of neurological deficits. Sequencing of cfDNA in the CSF revealed an H3 K27M alteration with a VAF of 58.82% (Figure 3B; Figure S4). This alteration was not detected in the blood. Given the imaging findings and the sequencing results, after interdisciplinary discussion, a diffuse midline glioma, H3 K27M altered, was the preferred diagnosis, and the patient underwent further treatment accordingly.

## Discussion

Here, we report on the feasibility of applying an NGS-based gene panel for sequencing cfDNA from CSF and blood of brain tumor patients to reveal cancer-specific mutations. Compared to sequencing from blood, sequencing cfDNA from CSF was superior in detecting more mutations in patients with brain cancer, as reported in previous work.^14^

The proximity of brain tumors to the CSF relative to blood could be 1 reason accounting for this. The blood–brain barrier might hinder the transfer of cfDNA into the bloodstream. The success rate of 55% of CSF samples is in the range of what others have reported.^9^^,^^15^ We showed that successful sequencing in CSF was associated with a larger tumor size and a shorter lesion distance to the CSF.^16-19^ However, regarding the tumor size, there are conflicting data.^7^ How tumor size, distance to CSF, and other factors affect NGS performance needs to be characterized in future large prospective clinical trials. Alterations that were detected exclusively in blood but were absent in matched tissue and CSF might originate from clonal hematopoiesis.^20^

Low cfDNA levels were postulated in other studies as the reason for the failure of analysis.^9^ More sensitive NGS panels have been developed to overcome low-input limitations.^8^ Here, the total amount of extracted cfDNA did not differ significantly between successfully sequenced and failed groups. Interestingly, a significant divergence in DNA yield after hybrid capture, with a trend already observable after library preparation, might indicate that the quality and integrity of cfDNA fragments might have influenced sequencing outcomes. As observed in our fragment size distribution analysis, blood-derived cfDNA showed a single peak at 167 bp, consistent with mononucleosomal DNA, while CSF cfDNA exhibited a broader range of fragment sizes, with a peak at 167 bp but additional smaller peaks (Figure S2). These differences in fragmentation patterns between blood and CSF align well with results from previous works on CSF obtained from lumbar puncture.^21^ Smaller or degraded cfDNA fragments may lead to reduced adapter ligation efficiency or lower PCR amplification efficiency. This could explain why DNA yield was significantly lower in failed sequencing samples after hybrid capture, even though extracted cfDNA amounts were similar across groups. Optimized low-input library protocols might improve the sequencing success rate. One further potential reason could be the abundance of cfDNA from healthy cells in blood, which may contribute to sequencing success while being non-informative compared to CSF in brain cancer patients. Further analysis to clarify the causes of these differences in sequencing success is needed.

While tumor-derived cfDNA in both blood and CSF is known to exhibit a different size distribution compared to non-tumor cfDNA, the exact mechanism that leads to cfDNA fragmentation is unclear.^22-24^ In previous work, mutations were more frequently detected in samples obtained via ventriculostomy compared to those collected by lumbar puncture.^9^ For larger tumors, lesions close to the ventricles, and when sampling near the time of surgery, more intact tumor DNA might reach the lumbar cistern, leading to higher detection rates.^25^ Beyond DNA properties, pre-analytical factors such as sample handling and storage or variability in lumbar puncture technique may have contributed to sequencing failure. Nevertheless, CSF is a low-protein matrix and therefore conveys lower potential interacting biomolecules than, for example blood, which leads to higher stability of several markers in CSF.

We tested the targeted sequencing of cfDNA in CSF and blood in a clinical setting. Thus, some cases turned out to be non-neoplastic (*N* = 3). For other patients, the final diagnosis is still pending due to a watch-and-wait strategy or inconclusive results. Nonetheless, we demonstrated that the proposed approach could add value to the diagnostic steps by revealing cancer-specific mutations, consistent with previous findings.^26-28^ In 2 cases, this led, combined with the imaging findings and after interdisciplinary discussion, to the diagnosis of a primary central nervous system lymphoma (PCNSL) and a diffuse midline glioma, H3 K27M-altered, respectively. In addition, we also detected a *BRAF* V600E mutation in the CSF of 1 glioblastoma patient, which is a druggable alteration.^29^ The ability to non-invasively detect actionable mutations could enhance treatment planning.

Major limitations of this study include the small sample size, heterogeneity of diagnoses, and enrichment for metastatic, non-CNS primary tumors. Further studies on homogeneous populations are necessary to validate clinical utility and to determine diagnostic accuracy. The NGS panel used in this study is not validated for CSF. Therefore, a larger cohort is needed in future studies to determine sensitivity, specificity, and limit of detection for sequencing of cfDNA in CSF. Moreover, matched sequencing of leukocyte-derived DNA should be performed in future studies to filter out mutations derived from clonal hematopoiesis.

Additionally, while our sequencing panel covered a broad range of oncogenic mutations, it is possible that other relevant genetic alterations were missed due to panel design constraints. In summary, NGS-based cfDNA sequencing in CSF should be considered if the cerebral lesion is close to the CSF. Moreover, in cases where invasive procedures such as biopsies pose a high risk, cfDNA sequencing from CSF may serve as a valuable alternative.

## Supplementary Material

vdaf270_Supplementary_Data

## Data Availability

The deidentified sequencing data generated in this study are shown in Table S2 filtered for pathogenic and likely pathogenic alterations.

## References

[1] Louis DN , Perry A, Wesseling P, et al. The 2021 WHO classification of tumors of the central nervous system: a summary. Neuro Oncol. 2021;23:1231-1251. 10.1093/neuonc/noab106 34185076 PMC8328013

[2] Malone H , Yang J, Hershman DL, Wright JD, Bruce JN, Neugut AI. Complications following stereotactic needle biopsy of intracranial tumors. World Neurosurg. 2015;84:1084-1089. 10.1016/j.wneu.2015.05.025 26008141

[3] Corcoran RB , Chabner BA. Application of cell-free DNA analysis to cancer treatment. N Engl J Med. 2018;379:1754-1765. 10.1056/NEJMra1706174 30380390

[4] Crucitta S , Pasqualetti F, Gonnelli A, et al. IDH1 mutation is detectable in plasma cell-free DNA and is associated with survival outcome in glioma patients. BMC Cancer. 2024;24:31. 10.1186/s12885-023-11726-0 38172718 PMC10763009

[5] Cantor E , Wierzbicki K, Tarapore RS, et al. Serial H3K27M cell-free tumor DNA (cf-tDNA) tracking predicts ONC201 treatment response and progression in diffuse midline glioma. Neuro Oncol. 2022;24:1366-1374. 10.1093/neuonc/noac030 35137228 PMC9340643

[6] Gupta M , Bradley JD, Massaad E, et al. Rapid tumor DNA analysis of cerebrospinal fluid accelerates treatment of central nervous system lymphoma. Blood. 2024;144:1093-1100. 10.1182/blood.2024023832 38776489 PMC11406186

[7] Iser F , Hinz F, Hoffmann DC, et al. Cerebrospinal fluid cfDNA sequencing for classification of central nervous system glioma. Clin Cancer Res. 2024;30:2974-2985. 10.1158/1078-0432.CCR-23-2907 38295147 PMC11247324

[8] Miller AM , Szalontay L, Bouvier N, et al. Next-generation sequencing of cerebrospinal fluid for clinical molecular diagnostics in pediatric, adolescent and young adult brain tumor patients. Neuro Oncol. 2022;24:1763-1772. 10.1093/neuonc/noac035 35148412 PMC9527510

[9] Ronsley R , Karvonen KA, Cole B, et al. Detection of tumor-derived cell-free DNA in cerebrospinal fluid using a clinically validated targeted sequencing panel for pediatric brain tumors. J Neurooncol. 2024;168:215-224. 10.1007/s11060-024-04645-y 38755519 PMC13440093

[10] Pan C , Diplas BH, Chen X, et al. Molecular profiling of tumors of the brainstem by sequencing of CSF-derived circulating tumor DNA. Acta Neuropathol. 2019;137:297-306. 10.1007/s00401-018-1936-6 30460397 PMC7523750

[11] McEwen AE , Leary SES, Lockwood CM. Beyond the blood: CSF-derived cfDNA for diagnosis and characterization of CNS tumors. Front Cell Dev Biol. 2020;8:45. 10.3389/fcell.2020.00045 32133357 PMC7039816

[12] Woodhouse R , Li M, Hughes J, et al. Clinical and analytical validation of FoundationOne Liquid CDx, a novel 324-Gene cfDNA-based comprehensive genomic profiling assay for cancers of solid tumor origin. PLoS One. 2020;15:e0237802. 10.1371/journal.pone.0237802 32976510 PMC7518588

[13] Shah M , Takayasu T, Zorofchian Moghadamtousi S, et al. Evaluation of the oncomine pan-cancer cell-free assay for analyzing circulating tumor DNA in the cerebrospinal fluid in patients with central nervous system malignancies. J Mol Diagn. 2021;23:171-180. 10.1016/j.jmoldx.2020.10.013 33531134 PMC7874332

[14] De Mattos-Arruda L , Mayor R, Ng CKY, et al. Cerebrospinal fluid-derived circulating tumour DNA better represents the genomic alterations of brain tumours than plasma. Nat Commun. 2015;6:8839. 10.1038/ncomms9839 26554728 PMC5426516

[15] Miller AM , Shah RH, Pentsova EI, et al. Tracking tumour evolution in glioma through liquid biopsies of cerebrospinal fluid. Nature. 2019;565:654-658. 10.1038/s41586-019-0882-3 30675060 PMC6457907

[16] Ohira T , Sakai K, Matsubayashi J, et al. Tumor volume determines the feasibility of cell‐free DNA sequencing for mutation detection in non‐small cell lung cancer. Cancer Sci. 2016;107:1660-1666. 10.1111/cas.13068 27575703 PMC5132294

[17] Chai R , An S, Lin H, et al. Sequencing of cerebrospinal fluid cell-free DNA facilitated early differential diagnosis of intramedullary spinal cord tumors. NPJ Precis Oncol. 2024;8:43. 10.1038/s41698-024-00541-w 38388726 PMC10884012

[18] Li M , Hou X, Zheng L, et al. Utilizing phenotypic characteristics of metastatic brain tumors to improve the probability of detecting circulating tumor DNA from cerebrospinal fluid in non-small-cell lung cancer patients: development and validation of a prediction model in a prospective cohort study. ESMO Open. 2022;7:100305. 10.1016/j.esmoop.2021.100305 34922300 PMC8685990

[19] Orzan F , De Bacco F, Lazzarini E, et al. Liquid biopsy of cerebrospinal fluid enables selective profiling of glioma molecular subtypes at first clinical presentation. Clin Cancer Res. 2023;29:1252-1266. 10.1158/1078-0432.ccr-22-2903 36648487 PMC10068436

[20] Okamura R , Piccioni DE, Boichard A, et al. High prevalence of clonal hematopoiesis‐type genomic abnormalities in cell‐freeDNAin invasive gliomas after treatment. Int J Cancer. 2021;148:2839-2847. 10.1002/ijc.33481 33497479 PMC8048515

[21] Mouliere F , Mair R, Chandrananda D, et al. Detection of cell‐free DNA fragmentation and copy number alterations in cerebrospinal fluid from glioma patients. EMBO Mol Med. 2018;10:e9323. 10.15252/emmm.201809323 30401727 PMC6284385

[22] Zheng YWL , Chan KCA, Sun H, et al. Nonhematopoietically derived DNA is shorter than hematopoietically derived DNA in plasma: a transplantation model. Clin Chem. 2012;58:549-558. 10.1373/clinchem.2011.169318 22052939

[23] Kustanovich A , Schwartz R, Peretz T, Grinshpun A. Life and death of circulating cell-free DNA. Cancer Biol Ther. 2019;20:1057-1067. 10.1080/15384047.2019.1598759 30990132 PMC6606043

[24] Mouliere F , Chandrananda D, Piskorz AM, et al. Enhanced detection of circulating tumor DNA by fragment size analysis. Sci Transl Med. 2018;10:eaat4921. 10.1126/scitranslmed.aat4921 30404863 PMC6483061

[25] Henriksen TV , Reinert T, Christensen E, et al.; IMPROVE Study Group. The effect of surgical trauma on circulating free DNA levels in cancer patients—implications for studies of circulating tumor DNA. Mol Oncol. 2020;14:1670-1679. 10.1002/1878-0261.12729 32471011 PMC7400779

[26] Ferreri AJM , Calimeri T, Lopedote P, et al. *MYD88* L265P mutation and interleukin‐10 detection in cerebrospinal fluid are highly specific discriminating markers in patients with primary central nervous system lymphoma: results from a prospective study. Br J Haematol. 2021;193:497-505. 10.1111/bjh.17357 33620087

[27] Wang Q , Liang Q, Wei W, et al. Concordance analysis of cerebrospinal fluid with the tumor tissue for integrated diagnosis in gliomas based on next-generation sequencing. Pathol Oncol Res. 2023;29:1611391. 10.3389/pore.2023.1611391 37822669 PMC10562547

[28] Pieri V , Curti DG, Paterra R, et al. CSF-based liquid biopsy pointing to a diagnosis of diffuse glioma in a patient with supposed neurodegenerative disorder. Neurol Sci. 2023;44:3271-3277. 10.1007/s10072-023-06806-9 37067723

[29] Subbiah V , Kreitman RJ, Wainberg ZA, et al. Dabrafenib plus trametinib in BRAFV600E-mutated rare cancers: the phase 2 ROAR trial. Nat Med. 2023;29:1103-1112. 10.1038/s41591-023-02321-8 37059834 PMC10202803

