# Supplementary material for: Targeted next-generation sequencing-based sequencing of cell-free DNA in cerebrospinal fluid uncovers cancer-specific mutations in patients with brain cancer using a widely available panel
Source: Neurooncol Adv. 2026 Jan 7;8(1):vdaf270. doi: 10.1093/noajnl/vdaf270 (PMC12883210; doi:10.1093/noajnl/vdaf270)
Supplement: vdaf270_Supplementary_Data [file vdaf270_supplementary_data.zip › Supplementary_material.docx]

Supplementary material

Figure S1. **Diagnostic performance by sample type and ancillary studies.** **A:** left: DNA yield in ng after library construction in the NGS successful and NGS failed groups (p = 0.056). Right: DNA yield in ng after hybrid capture in ng in these two groups (p = 0.003). **B:** Representative measurement of the product of diameter (POD). **C:** Glucose ratios (CSF/serum, p = 0.25), lactate levels in mmol/l (p = 0.19), and albumin ratios (p = 0.11) are shown.

Figure S2. **cfDNA fragment length distribution in bp**

Distribution of cfDNA length in bp isolated from blood (upper plot) and CSF (lower plot).

Figure S3. **Results of targeted NGS in CSF and blood using F1LCDx in patient 19 (CSF results are Research Use Only)**

All pathogenic mutations and VUS from CSF compared to blood from patient 19.

Figure S4. **Results of targeted NGS in CSF and blood using F1LCDx in patient 36 (CSF results are Research Use Only)**

All pathogenic mutations and VUS from CSF compared to blood from patient 36.

Table S1. **Overview of measurements**

Overview of analysis including CSF volume, POD, tumor size, CSF chemistry, albumin ratio, total albumin, DNA yield after isolation, library construction (LC), hybrid capture (HC). Status analysis “0” corresponds to a failed sequencing analysis whereas “1” means successful analysis.

Table S2. **Overview of pathogenic and likely pathogenic variants**
