# Supplementary figures and images for: Targeted next-generation sequencing-based sequencing of cell-free DNA in cerebrospinal fluid uncovers cancer-specific mutations in patients with brain cancer using a widely available panel
Source: Neurooncol Adv. 2026 Jan 7;8(1):vdaf270. doi: 10.1093/noajnl/vdaf270 (PMC12883210; doi:10.1093/noajnl/vdaf270)

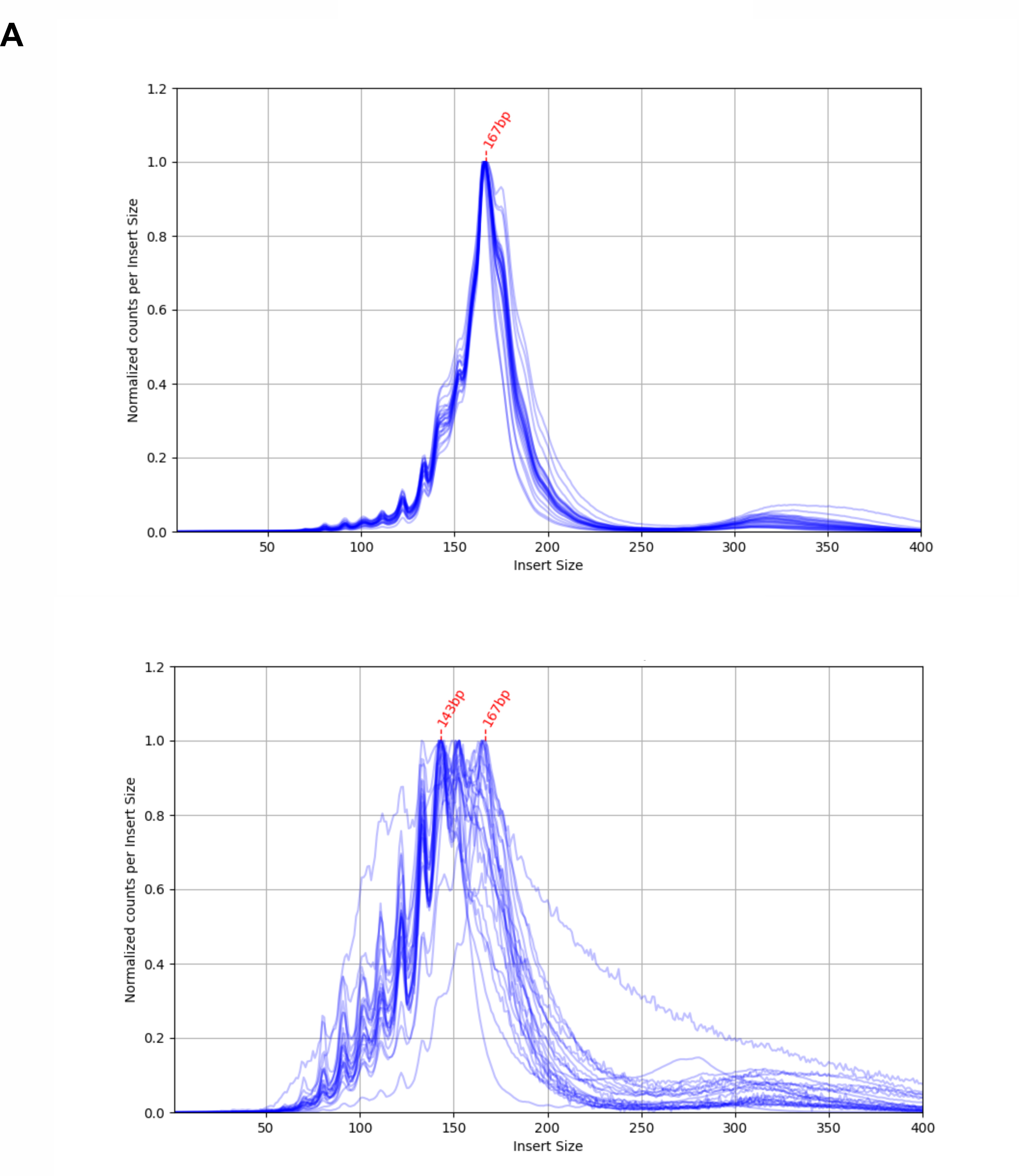

Supplement: vdaf270_Supplementary_Data [file vdaf270_supplementary_data.zip › Figure_S2.tiff]

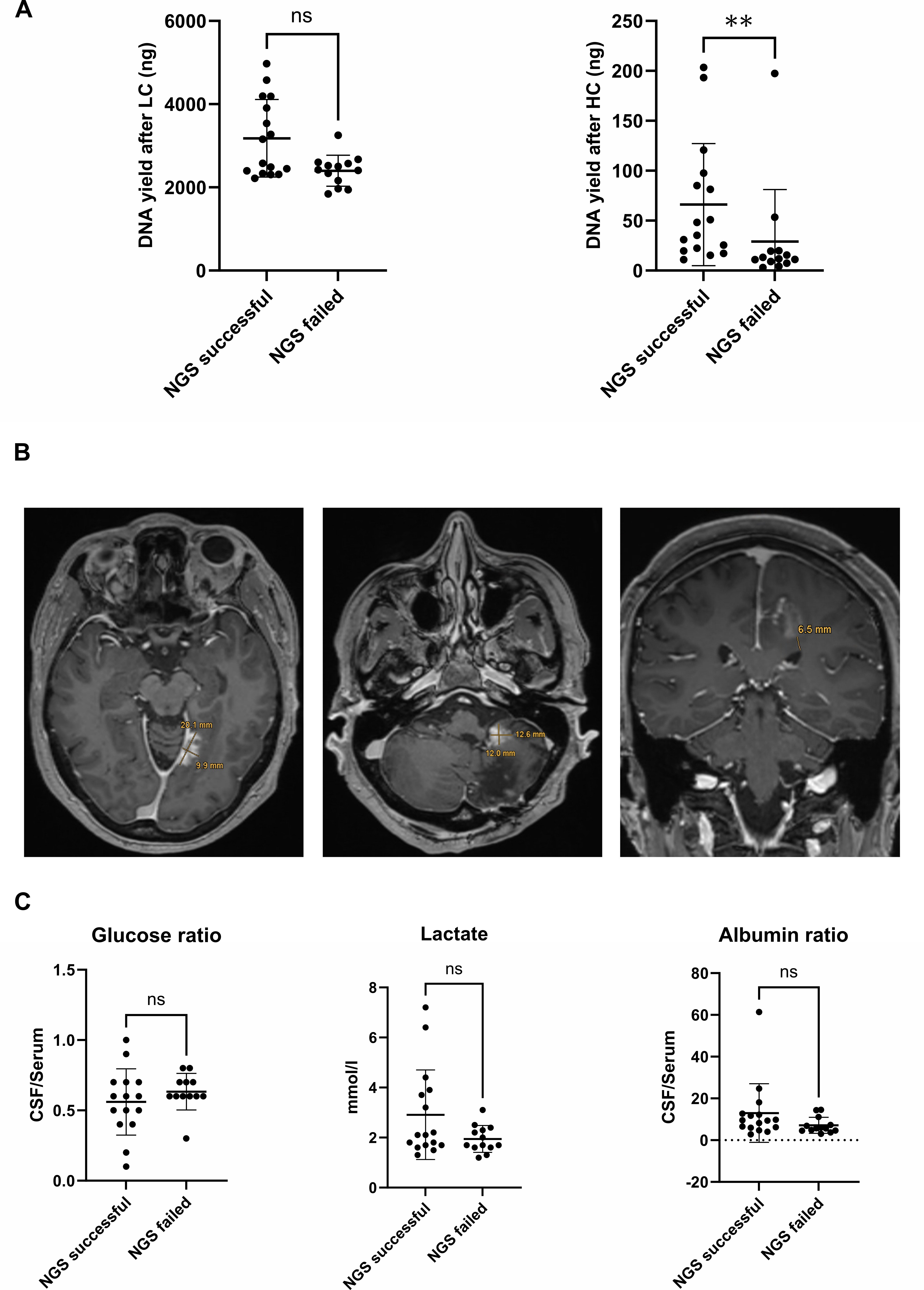

Supplement: vdaf270_Supplementary_Data [file vdaf270_supplementary_data.zip › Figure_S1.tiff]

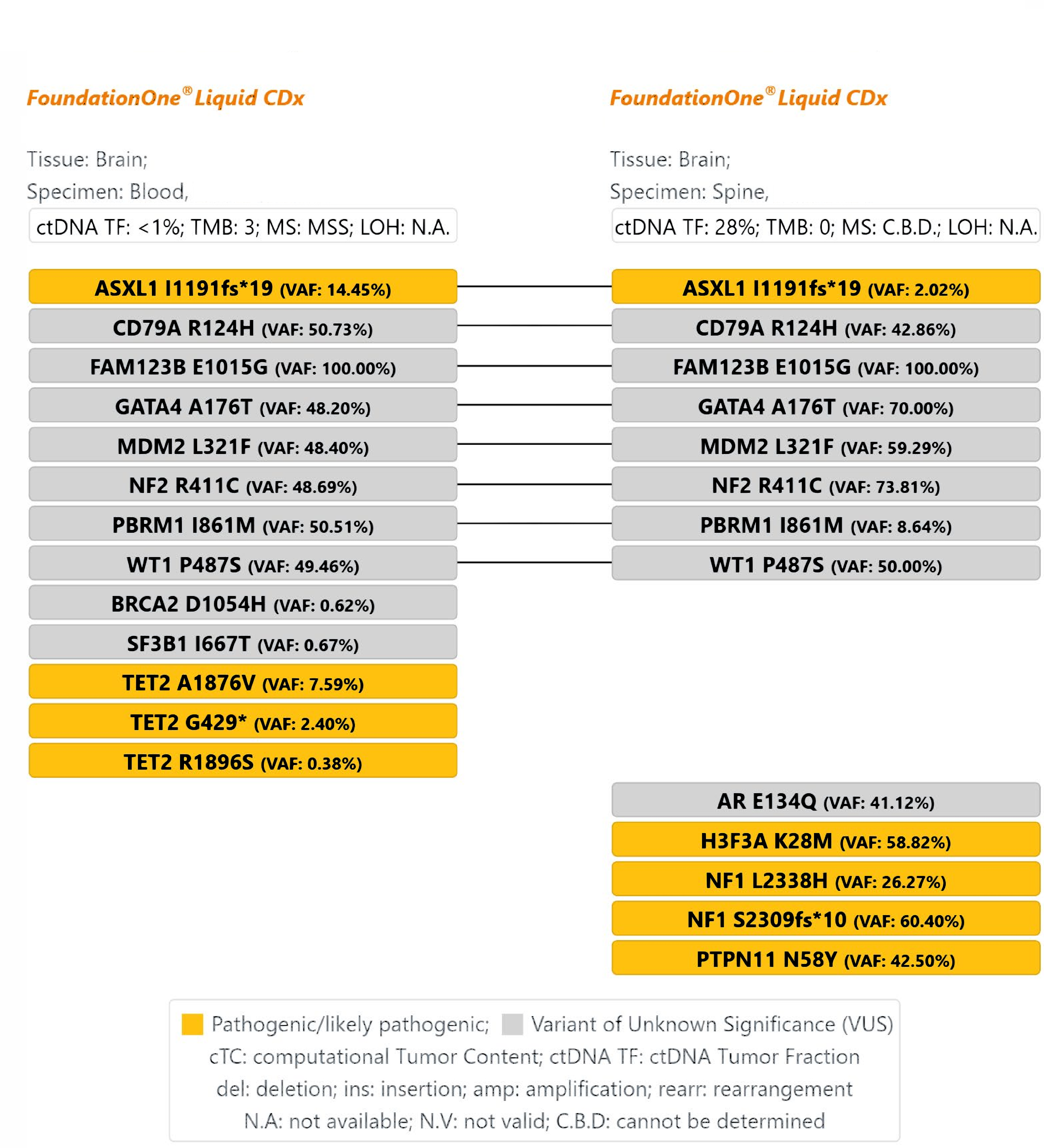

Supplement: vdaf270_Supplementary_Data [file vdaf270_supplementary_data.zip › Figure_S4.tiff]

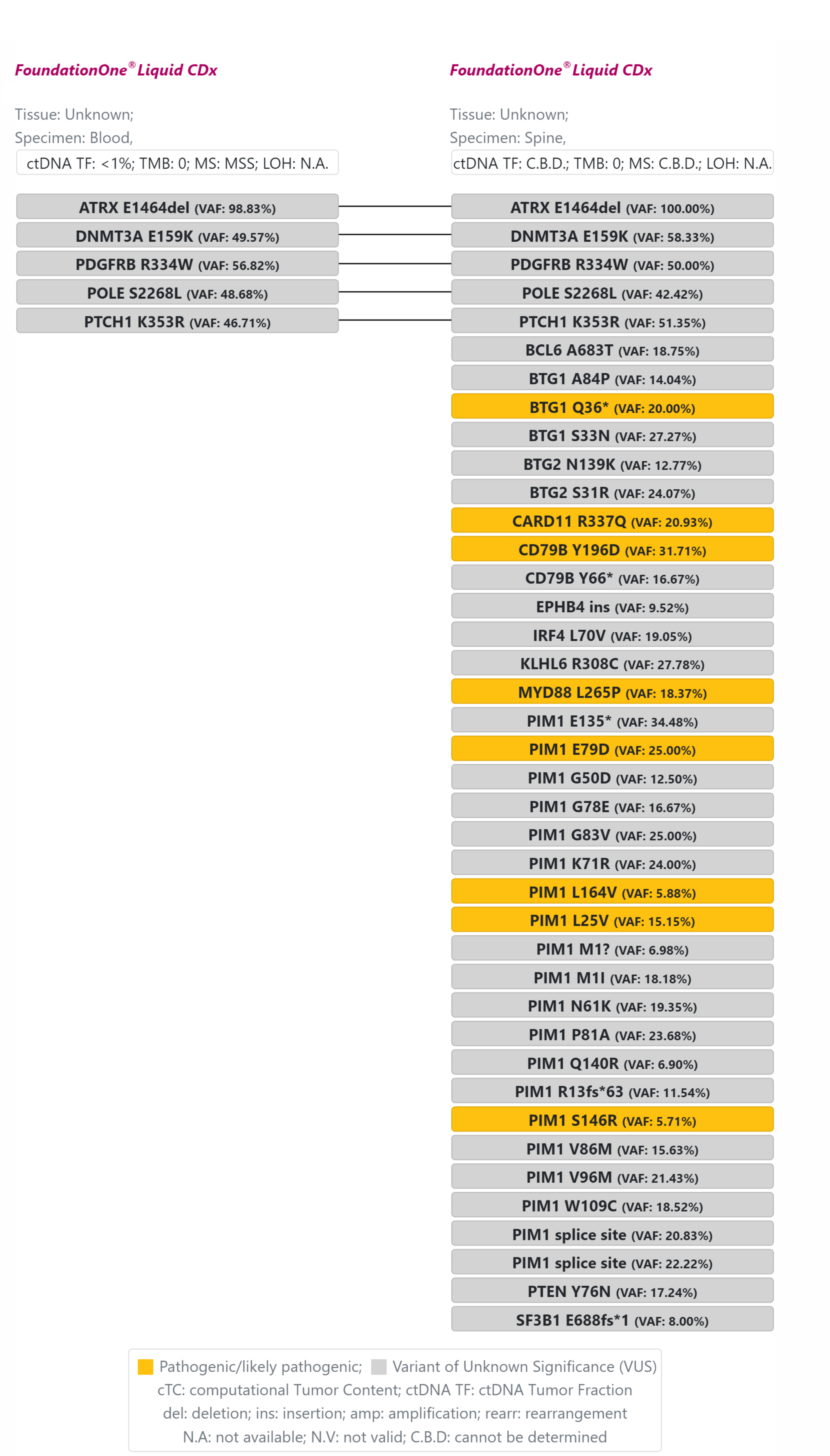

Supplement: vdaf270_Supplementary_Data [file vdaf270_supplementary_data.zip › Figure_S3.tiff]
